# Automated facial expression measurement in a longitudinal sample of 4- and 8-month-olds: Baby FaceReader 9 and manual coding of affective expressions

**DOI:** 10.3758/s13428-023-02301-3

**Published:** 2024-01-25

**Authors:** Martina S. Zaharieva, Eliala A. Salvadori, Daniel S. Messinger, Ingmar Visser, Cristina Colonnesi

**Affiliations:** 1 Department of Developmental Psychology, Faculty of Social and Behavioural Sciences, University of Amsterdam, Nieuwe Achtergracht 129b, 1001 NK Amsterdam, The Netherlands; 2 Developmental Psychopathology Unit, Development and Education, Faculty of Social and Behavioural Sciences, Research Institute of Child, University of Amsterdam, Nieuwe Achtergracht 129b, 1001 NK Amsterdam, The Netherlands; 3 Yield, Research Priority Area, University of Amsterdam, Amsterdam, The Netherlands; 4 Department of Psychology, University of Miami, Coral Gables, FL USA; 5 Department of Pediatrics, University of Miami, Coral Gables, FL USA; 6 Department of Music Engineering, University of Miami, Coral Gables, FL USA; 7 Department of Electrical and Computer Engineering, University of Miami, Coral Gables, FL USA

**Keywords:** Baby FaceReader 9, Automated facial expression measurement, Manual micro-coding, Between-system agreement, Infant, Face-to-face interaction

## Abstract

Facial expressions are among the earliest behaviors infants use to express emotional states, and are crucial to preverbal social interaction. Manual coding of infant facial expressions, however, is laborious and poses limitations to replicability. Recent developments in computer vision have advanced automated facial expression analyses in adults, providing reproducible results at lower time investment. Baby FaceReader 9 is commercially available software for automated measurement of infant facial expressions, but has received little validation. We compared Baby FaceReader 9 output to manual micro-coding of positive, negative, or neutral facial expressions in a longitudinal dataset of 58 infants at 4 and 8 months of age during naturalistic face-to-face interactions with the mother, father, and an unfamiliar adult. Baby FaceReader 9’s global emotional valence formula yielded reasonable classification accuracy (*AUC* = .81) for discriminating manually coded positive from negative/neutral facial expressions; however, the discrimination of negative from neutral facial expressions was not reliable (*AUC* = .58). Automatically detected a priori action unit (AU) configurations for distinguishing positive from negative facial expressions based on existing literature were also not reliable. A parsimonious approach using only automatically detected smiling (AU12) yielded good performance for discriminating positive from negative/neutral facial expressions (*AUC* = .86). Likewise, automatically detected brow lowering (AU3+AU4) reliably distinguished neutral from negative facial expressions (*AUC* = .79). These results provide initial support for the use of selected automatically detected individual facial actions to index positive and negative affect in young infants, but shed doubt on the accuracy of complex a priori formulas.

Facial expressions are crucial to preverbal social interaction and among the earliest behaviors that can be used for inferring emotional states in infants (e.g., Bolzani et al., 2002; Messinger, 2002; Oster et al., 1992; Oster, 2003, 2005a, b). The systematic classification and tracking of facial expressions during infant–caregiver interactions has been invaluable for studying a wide range of early socio-cognitive and socio-emotional developments, including the emergence and dynamics of visual attention (e.g., Hietanen & Leppänen, 2003; Lavelli & Fogel, 2005), emotion regulation (e.g., MacLean et al., 2014; Mangelsdorf et al., 1995), and preverbal communication (e.g., Beebe et al., 2016; Colonnesi et al., 2012; Hsu & Fogel, 2001; Yale et al., 1999, 2003).

The manual coding of facial expressions is a labor-intensive process, however, and procedural variations along with subjective factors may limit the reproducibility of results obtained at different infant labs. Recent developments in computer vision have brought about substantial advances in automated facial expression recognition in adult data, offering the potential for a powerful and relatively lower time-investment alternative to manual behavioral coding (e.g., Ertugrul et al., 2019; Niinuma et al., 2019; Yang et al., 2019). The application of automated measurement methods to infant facial expression data could sustain rich analysis of facial behavior and at the same time increase the replicability of studies involving infant behavior by allowing researchers to apply objective measures to much larger sample sizes than what is typically feasible with manual coding techniques. Before the use of such methods can be reliably introduced in infant research, however, we first need to assess whether available automated facial expression detection systems can produce results that are comparable to those obtained from manual human coding. The current work provides a detailed performance evaluation of one automated system – Baby FaceReader 9 (Noldus, 2022), comparing it to manual coding of affective facial expressions in a longitudinal dataset of infants at 4 and 8 months of age during naturalistic face-to-face interactions.

## Manual coding of affective facial expressions

Manual coding techniques have a long-standing tradition in developmental research, and coding systems vary substantially in terms of the extent to which affective labels are used to describe facial behavior (Cohn & Ekman, 2005; Cohn et al., 2007; Harrigan, 2013; Stern, 1971). The most comprehensive coding system available is the Baby Action Coding System (Baby FACS; Oster, 2006), which involves the systematic, anatomically based classification of the frequency and duration of activation across discrete facial muscle action units (AUs). Configurations of action units and their intensities can then serve for inferring discrete affective states while taking into account infant–adult differences in facial morphology (Oster & Ekman, 1978). Thus, unlike other approaches, the inferences made about the affective meaning of facial expressions are extrinsic to the coding system, which limits the involvement of subjective judgment during the coding process and makes results more likely to be reproduced (Cohn et al., 2007; Oster et al., 1992).

Baby FACS requires coders to undergo an extensive training certification and therefore alternative, less involved coding systems for coding affective facial expressions in more holistic terms are frequently employed throughout developmental research. A common approach is, for instance, to track second-by-second changes in the global emotional valence of facial expressions (e.g., Aktar et al., 2017; Colonnesi et al., 2012; Salvadori et al., 2021, 2022). Infants’ facial states are coded into discrete categories: positive facial expressions (i.e., smiles), negative facial expressions (i.e., frowns, pouting or lip stretching), or neutral (i.e., absence of positive and negative facial expressions). Facial expressions in each valence category are typically analyzed in terms of durations or event frequencies. Common to most if not all manual coding techniques is that the reliability of the coding is assessed using inter-rater reliability metrics among at least two trained independent raters. A specific characteristic of such manual coding approaches is that they are based on the subjective detection of facial expressions within a specific interaction context (e.g., the infants’ general affective state during the interaction, contextual information, reaction of the social partner), and that the intensity of the affective state is typically not coded (but cf. Beebe et al., 2009; 2010; Kokkinaki, 2009). Because such approaches are frequently used across developmental science domains (e.g., Feldman, 2007; Leclére et al., 2014), and the proportion of time or temporal change of the facial expression’s valence tend to be the primary outcome variables researchers are after, we chose to focus our analysis on a dataset that is comparable in those respects to other observational infant research.

## Automated measurement tools for infant facial expressions

Applying adult models to infant data is problematic because infant facial features differ substantially from those of adult’s (Ertugrul et al., 2023; Oster, 2005a, b, 2006; Oster & Ekman, 1978) and publicly available benchmark datasets for training automated facial expression analysis algorithms are limited (but cf. Messinger, 2014; Nanni et al., 2010; Webb et al., 2018). Several open-source tools have shown promising results in comparison to manual coding (e.g., Ertugrul et al., 2023; Hammal et al., 2017; Messinger et al., 2009; 2012; Zamzmi et al., 2017). A main hurdle for popularizing such approaches in developmental research, however, is that the use of such tools requires a variety of technical skills. Baby FaceReader 9 (Noldus, 2022) is a commercially available solution that is marketed for automated measurement of infant facial expressions, and offers a graphical user interface, which makes it suitable for research teams with little computer vision or programming expertise.

### Previous validation work using Baby FaceReader

Previous validation work on Baby FaceReader’s performance is limited. Baby FaceReader 8 (Noldus, 2016) has been applied in two small-scale studies from conference posters to**:** 1) detect manually annotated facial action units associated with food preferences from 74 static images (age not publicly available; Maroulis et al., 2017), and 2) evaluate which automatically detected action units associate with manual codings of global emotional valence from image data of 0- to 12-month-old infants (Maroulis, 2018). Preliminary results from Maroulis and colleagues (2017) showed classification accuracy scores ranging between .24 and .80 against manually annotated action units. Baby FaceReader 9 (Noldus, 2022) is based on a deep convolutional neural network (CNN) classification approach that aims to improve the speed and accuracy of the face detection and modeling stages of Baby FaceReader 8, and no previous work has evaluated its performance compared to manually coded infant data. Since developmental research on young infants typically focuses on analyzing real-time facial expressions from naturalistic social interactions, we build on previous work by evaluating the results from Baby FaceReader 9 with respect to manually coded facial expression valence in a pre-existing longitudinal dataset of infant videos collected during home-based face-to-face interactions at 4 and 8 months of age. In the following, we briefly describe Baby FaceReader 9’s model architecture of and output.

### Automated measurement of facial expressions in Baby FaceReader 9

Baby FaceReader v.9.0.17 (Noldus, 2022) was developed for frame-rate automated measurement of affective facial expressions in infants between 6 and 24 months of age based on the Baby FACS manual coding system (Oster, 2006). Baby FaceReader 9 uses a deep learning-based approach (Zafeiriou et al., 2015) to localize a face in an image, and a deep convolutional neural network (CNN; Gudi et al., 2015) to detect action unit occurrence and estimate action unit intensity (Noldus, 2021; Noldus, personal communication, March 8, 2023). The CNN was trained on a compilation of publicly available and self-collected datasets, featuring spontaneous facial expressions of infants aged 6–24 months from multiple ethnic backgrounds and a roughly even sex distribution (Noldus, 2021; Noldus, personal communication, March 8, 2023). The resulting output is the continuous intensities of individual action units as defined in Oster’s Baby FACS (Noldus, 2021). The global emotional valence of the facial expression is computed based on specific action unit configurations reported in Maroulis (2018) (Noldus, 2021).

### Relating automatically detected action units to affective facial expressions

Several facial action units are known to show either selective or shared activation during the expression of positive and negative affect (Messinger et al., 2012; Oster et al., 1992; Oster, 2003, 2005a, b). We expected that the interaction effects among these action units would predict manual codings of positive and negative facial expressions (Table 1). Whereas Baby FaceReader 9 outputs continuous variables, manually coded data tends to be categorical. Here we explore the relations between manually coded facial expression categories and the activation intensities of automatically detected action unit configurations that have been previously reported to associate with positive and negative affective expressions.

**Table 1:** Expected main and interaction effects of action unit configurations predicting manual codings of positive versus negative facial expressions

Facial expression valence	Automatically detected action units
*Positive facial expressions*	Main effect of AU12
Smiles with eye constriction	AU12 * AU6
Smiles with mouth opening	AU12 * AU(25 + 26 + 27)
*Negative facial expressions*	Main effect of AU20
Pouting with brow lowering	AU17 * AU(3 + 4)
Lip stretching withbrow lowering	AU20 * AU(3 + 4)
Lip stretching with eye constriction	AU20 * AU(6 + 7)
Lip stretching with mouth opening	AU20 * AU(25 + 26 + 27)

Note. Configurations involving eye constriction [AU6] (referred to as “cheek raising” in the FACS manual; Ekman et al., 2002) are Duchenne expressions (i.e., Duchenne smiles and Duchenne cry-faces)

The prototypical expression of positive affect in infancy is through smiles, which are indexed by raising of the lip corners via the *zygomaticus major* (AU12) (Ekman et al., 2002; Messinger et al., 2001). Infant negative affect is predominantly displayed through frowns and cry-faces (Camras et al., 1992; Oster, 2003; Oster et al., 1992; Weinberg & Tronick, 1994), both indicated by stretching the lips via the *risorius* (AU20) (Messinger et al., 2012; Yale et al., 2003). Pouting may also involve lowered brows (*corrugator* *supercilii* [AU3] and/or *procerus* [AU4]) and displayed in combination with raised chin (*mentalis* [AU17]) during expressions of sadness (Bolzani Dinehart et al., 2005; Oster, 2006; Oster & Rosenstein, 1996; Yale et al., 2003).

Eye constriction and mouth opening appear to be associated with the intensity of infants’ affective expressions, independently predicting positive as well as negative facial expressions (Kohut et al., 2012; Fogel et al., 2006; Izard et al., 1987; Mattson et al., 2013; Messinger et al., 2009, 2012). Therefore, we expected that the action unit indices of eye constriction and mouth opening would predict manual codings of positive and negative facial expressions only when in configuration with other action units indexing emotional valence (Table 1). The mouth opening is displayed through a combination of the actions of *depressor* *labii* (AU25), *masetter* (AU26), and the *pterygoids* (AU27) (Messinger et al., 2012), whereas eye constriction in the prototypical cry-face is indicated through *orbicularis oculi pars orbitalis* (AU6) and *pars* *palpebralis* (AU7) (Izard et al., 1987; Kohut et al., 2012; Mattson et al., 2013).

## The current study

The goals of the current paper are: 1) to assess the construct validity and feasibility of Baby FaceReader 9’s global emotional valence formula by comparing it to manually coded infant facial expressions from naturalistic social interactions, 2) to explore the associations between a priori action unit configurations and manually coded facial expression categories, and 3) to explore a parsimonious approach assessing the association between individual action units and manually coded facial expression categories. To address these goals, we compared Baby FaceReader 9 and manually coded data in a pre-existing longitudinal dataset in which infants’ facial expressions during a face-to-face interaction were manually coded either as positive, negative, neutral (i.e., neither positive, nor negative), or not visible. Within-subject data were gathered with three interaction partners – mother, father, and an unfamiliar adult at 4 and 8 months of age. There was no overlap between the training data used for Baby FaceReader and the longitudinal dataset analyzed in the current study.

## Methods

### Video recordings

Data were obtained from a pre-existing longitudinal study (Salvadori et al., 2022), in which the facial expressions of 58 infants (25 female) were microcoded during home-based face-to-face interactions with three interaction partners (mother, father, an unfamiliar adult) at 4 and at 8 months. From the original sample, 36 of the observations were left without video data due to cancellation, three due to technical error, and 20 due to extreme fussiness. A total of 289 video observations remained available for the analysis: 51 infants at 4 months and 53 infants at 8 months. Participant descriptives as well as the number and durations of the video sample are shown in Table 2.

**Table 2:** Sample descriptives of the available video data

Descriptives	4 months	8 months
Participant *n*	51	53
*n* male	31	33
*n* female	20	20
*M* (*SD*) age in days	126.42 (7.93)	250.58 (9.67)
*n* videos	148	141
*n* mother	49	48
*n* father	50	46
*n* unfamiliar adult	49	47
*M* (*SD*) video duration in seconds	121.24 (5.81)	113.35 (20.98)

### Longitudinal study design and procedure

**Fig. 1:**
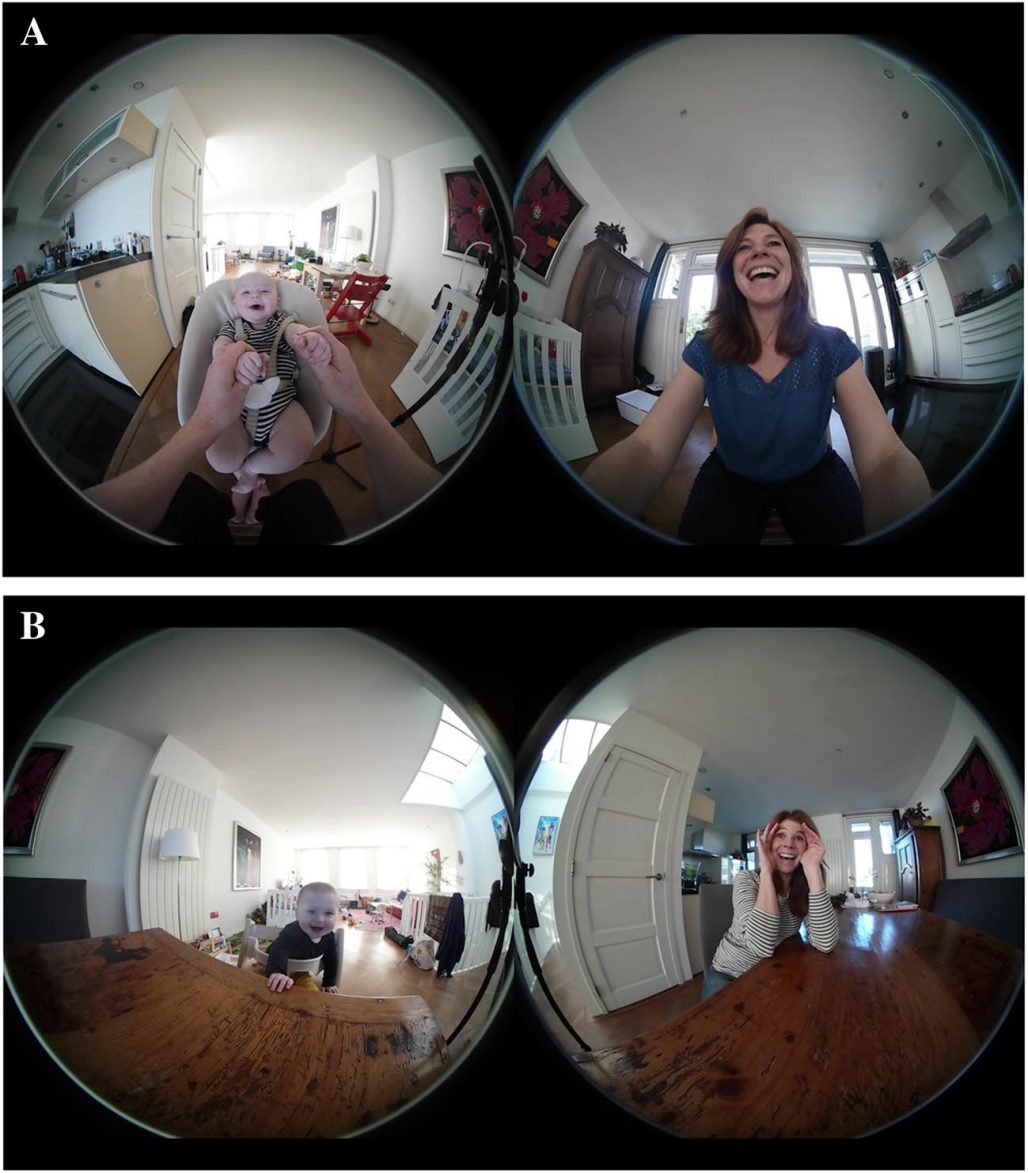
**A** Video recording setup at 4 months: Interaction view. **B **Video recording setup at 8 months: Interaction view. *Note.* Example of the raw video recording (3840 x 2160 pixels at 30 Hz) from a face-to-face interaction between a 4 and an 8-month-old infant and mother

At 4 and at 8 months, three 2-min naturalistic face-to-face interactions were recorded with each interaction partner: mother, father, and a (female) experimenter (Salvadori et al., 2022). The infant was positioned in an age-appropriate infant seat opposite the interaction partner as shown in Fig. 1A and B demonstrating the observational setup used for the same infant at 4 and at 8 months, respectively. Caregivers were instructed to interact with the infant as they would typically do in their everyday life. The order of the interaction partners was counterbalanced within infant sex across families. The observation was stopped if infants showed extreme distress.

### Video recording characteristics

**Fig. 2:**
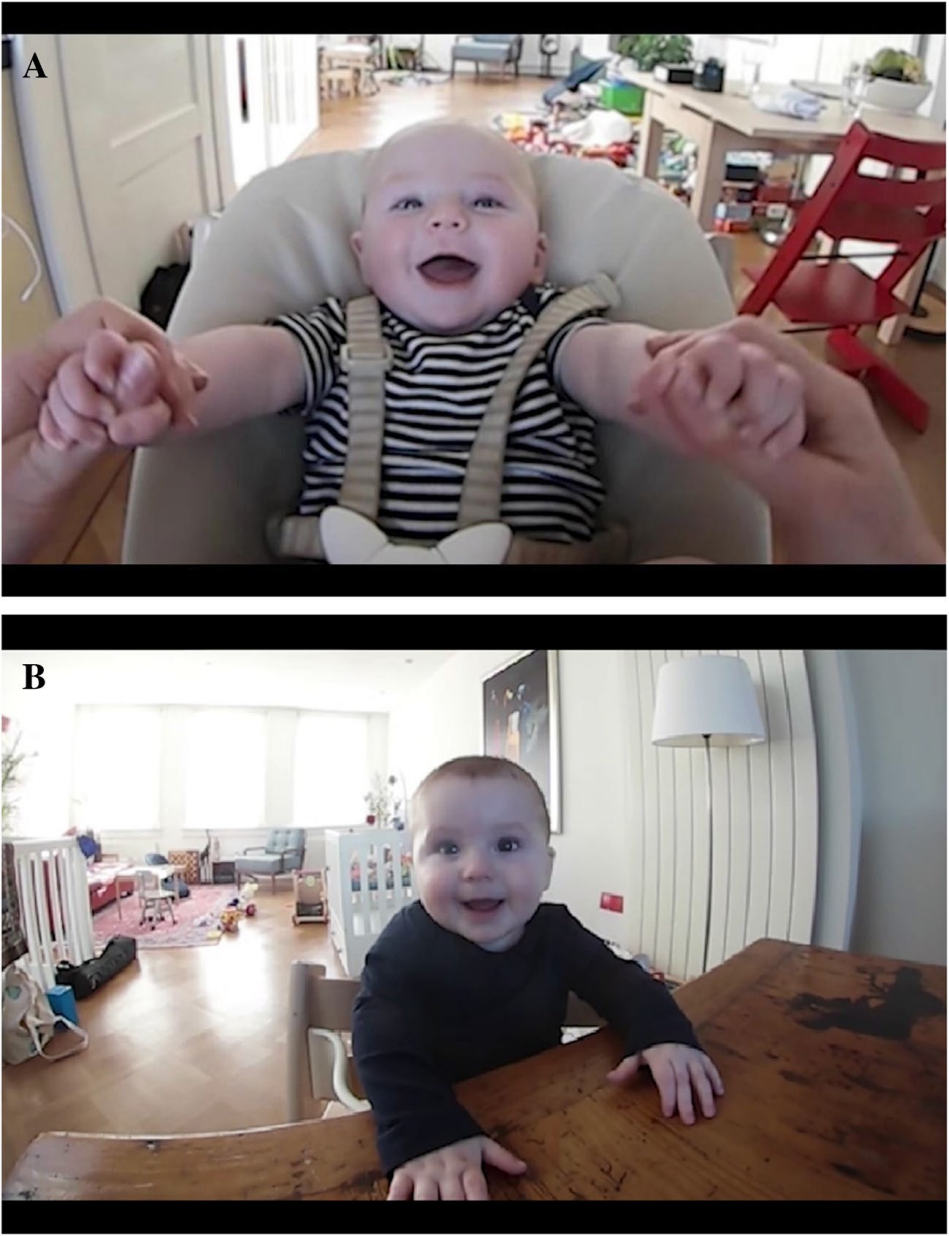
**A** Video recording setup at 4 months: Infant view. **B** Video recording setup at 8 months: Infant view. *Note.* Example of the video recording (1280 x 720 pixels at 30 Hz) of a 4 and an 8-month-old infant used for the manual and automated facial expression measurement in Baby FaceReader 9

The interactions were recorded using a mobile dual-lens camera (Samsung GEAR 360°, 2016) mounted between the infant and the adult, yielding a high-resolution wide-angle split-screen video recording (3840 x 2160 pixels at 30 Hz) of both interaction partners simultaneously (Fig. 1A, B). The same video recording zoomed in on the infant’s face and upper body (1280 x 720 pixels at 30 Hz) was used as input to the manual and automated measurement (Fig. 2A, B). A conservative estimate of the area of interest (AOI) of the infant face was derived from the distances between automatically registered 2D landmarks of the eyebrows and the mouth. The detailed computation and descriptives can be found in Supplementary Materials B (https://osf.io/7afmx). The mean face area fraction was .05 (M = 51,366 pixels, SD = 10,933) from the total image resolution (Table S3 [https://osf.io/7afmx]), which satisfies the minimum of .01 full face area fraction required by Baby FaceReader 9’s face localization algorithm. The mean face fraction was .02 higher at 4 compared to 8 months of age (Table S3 [https://osf.io/7afmx]), indicating shorter recording distance at 4 months.

### Manual coding of affective facial expressions

#### Coding scheme

Infants’ affective facial expressions were manually coded using The Observer XT 14.0 (Noldus et al., 2000; Zimmerman et al., 2009). Following Colonnesi et al. (2012), facial expressions were coded into one of four mutually exclusive categories: 1) *Positive* – involving Duchenne and non-Duchenne smiles (lip corner raising, AU12) with the mouth closed or open (AU25, AU26, AU27), with or without eye constriction indicated by cheek raising (AU6); 2) *Negative –* involving frowns, Duchenne and non-Duchenne pre-cry and cry-faces inferred from lowered-lip corners, constriction of the eye region, and opening of the mouth; 3) *Neutral –* when neither a positive nor a negative facial expression was displayed, i.e., either when no muscle movement was visible or the visible muscle movement was not indicative of an affective facial expression; 4) *Not visible* – when the face was occluded or out of focus. Note that manual coders had access to contextual information (e.g., a view of the interaction partner, sound) and were instructed to interpolate the previous facial state when brief facial occlusions were encountered.

#### Inter-rater reliability

The 289 video observations were coded by graduate students (five coders at the 4-month wave; three coders at the 8-month wave) that were trained on a subset of the videos by a senior coder until satisfactory inter-rater reliability (Cohen’s kappa > .70) was attained. Another 15% of the videos were randomly selected (counterbalanced within interaction partner, infant age, and sex) to be double coded by a senior coder (one of the co-authors). Inter-rater reliability for all manually coded facial expression categories was computed on the 15-Hz time samples of the double-coded data using the R-package “irr” (v. 0.84.1; Gamer et al., 2012), yielding a weighted kappa coefficient of .83 for the 4-month wave and .92 for the 8-month wave (Cohen, 1968). Inter-rater agreement on the percentage of time for which each facial expression category was coded was 93% and 95% at 4 and 8 months, respectively.

## Automated measurement of affective facial expressions

### Baby FaceReader 9 model architecture

Baby FaceReader v.9.0.17 (Noldus, 2022) was developed for frame-rate automated measurement of affective facial expressions in infants between 6 and 24 months of age based on the Baby FACS manual coding system (Oster, 2006). A face is located in the image using a deep learning-based face finding algorithm (Zafeiriou et al., 2015), which searches for areas in the image that have the appearance of a face at different scales (Noldus, 2021). A deep convolutional neural network (CNN; Gudi et al., 2015) compiles a 3D face model in a single pass by estimating the location of 468 facial landmarks (e.g., eye corner, lip corner, etc.) relative to learned landmark locations using a 2D grayscale pixel matrix of the face normalized for in-plane head rotations, scale, and global contrast (Noldus, 2021; Noldus, personal communication, March 8, 2023). The CNN was trained on a combination of real-world and synthetically generated faces to estimate manually labeled and auto-generated facial landmark locations in 3D space using a 3DMM face model (Bulat & Tzimiropoulos, 2017; Noldus, 2021). Depth (i.e., the distance of the face to the camera) is estimated from the camera’s parameters by comparing the scale of the face to a reference face scale (Noldus, personal communication, March 8, 2023). Facial landmarks are compressed into a vector representation using principal component analysis (Noldus, 2021). The CNN models underlying Baby FaceReader and FaceReader were simultaneously trained on action unit occurrences and Baby FACS intensity level categories (Ekman et al., 2002; Oster, 2006; Noldus, personal communication, March 8, 2023). Action unit occurrences and intensities are estimated directly from image pixels for each video frame using specific activation patterns of the output layer (Gudi et al., 2015; Noldus, 2021).

### Baby FaceReader 9 training dataset

The CNNs underlying Baby FaceReader 9 were trained on a compilation of publicly available and self-collected datasets involving spontaneous facial expressions of infants from multiple ethnicities in the age range of 6–24 months and roughly even sex distribution (Noldus, 2021; Noldus, personal communication, March 8, 2023). The data were collected primarily under lab settings with good lighting conditions and in the presence of a caregiver (Noldus, personal communication, March 8, 2023). Approximately 15,000 images were used for training; data augmentation was used to increase the effective number by an order of magnitude (Noldus, personal communication, March 8, 2023). The training set included largely frontal static images and video frames, with some containing pitch and yaw variations in the range of ± 30° angle with respect to the camera (Noldus, personal communication, March 8, 2023). Further details regarding the training datasets (e.g., sample size, age, sex, ethnic characteristics, observation scenarios) were not available.

### Automated measurement of positive and negative facial expressions

As indicated in the Baby FaceReader 9 user manual (Noldus, 2021), the raw action unit output represents continuous action unit intensities from 0 (low) to 1 (high) corresponding to the intensity categories described in Baby FACS (Oster, 2006): “inactive” [.00 – .10], A “trace” [.10 – .22]; B “slight ” [.22 – .33]; C “pronounced” [.33 – .62]; D “severe” [.62 – .91]; E “max” [.91 – 1.00] (Noldus, 2021). Additionally, the global emotional “valence” output summarizes the estimated affective intensity of the infant’s facial expression on a scale from – 1 (negative) to 1 (positive), where: 1) negative facial expressions take on negative valence values computed as the arithmetic mean across the intensity values of AU1 (inner brow raiser), AU3 and AU4 (brow lowering), AU7 (lid tightener), AU20 (lip stretching), AU25 (lips parting), and AU43 (eyes closed), and 2) positive facial expressions take on positive valence values computed as the arithmetic mean across the intensity values of AU6 (cheek raiser), AU12 (lip corner raiser), and AU25 (lips parting) (Noldus, 2021). Smooth classification (i.e., a recency-weighted moving average) was applied to reduce noise in sample-to-sample action unit intensity estimation.

### Missing data

**Fig. 3:**
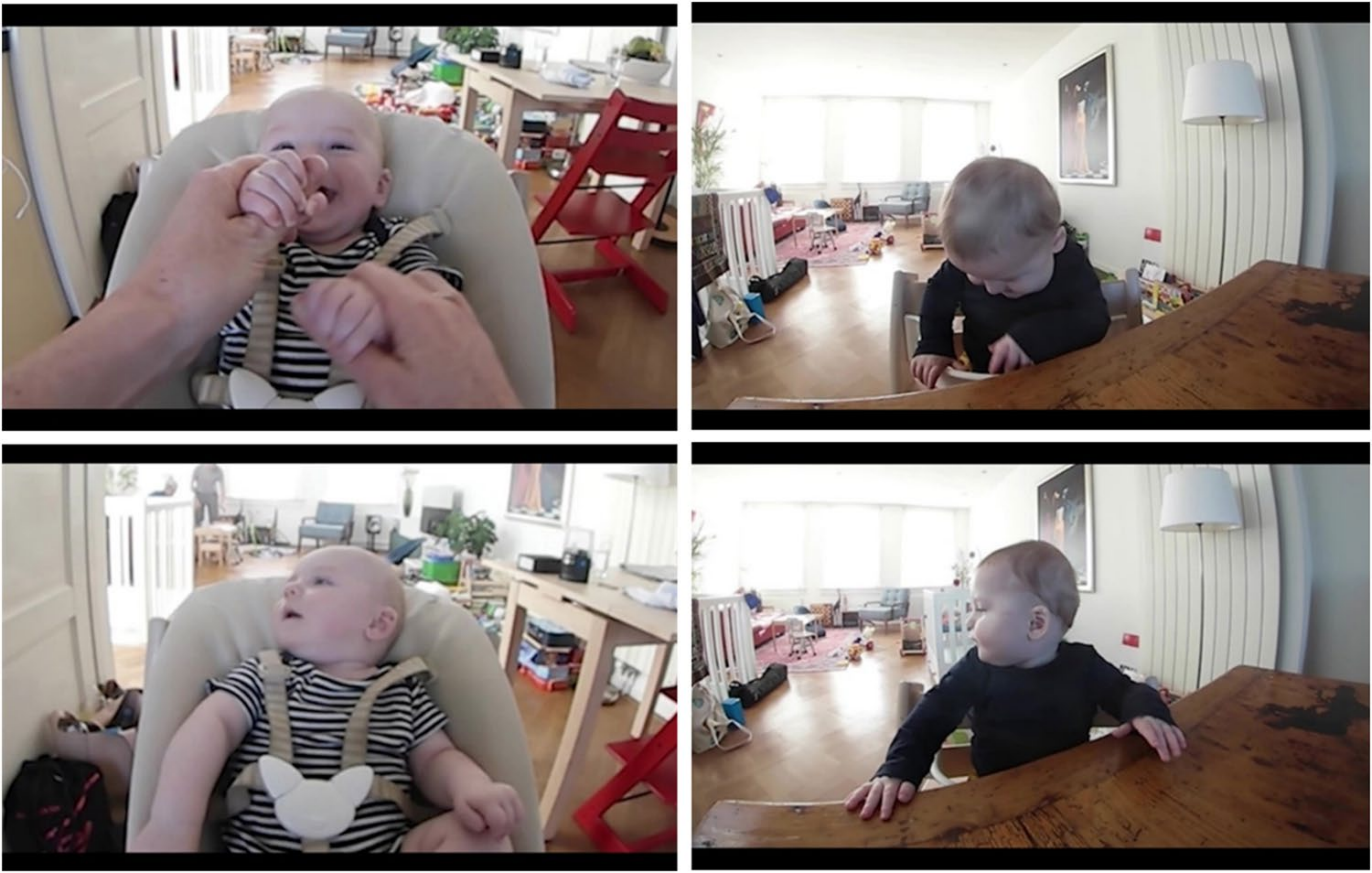
Partial facial occlusions causing missing data in automated action unit detection. *Note.* Partial facial occlusions encountered in the dataset from the same infant at 4 months (left top and bottom images) and at 8 months (right top and bottom images). Automated action unit detection failed for all frames presented, whereas manually coded affective facial expressions were available

Two sources of missing data are output by Baby FaceReader 9: 1) failure to detect the face (i.e., “detection failed”), and 2) failure to generate a face state model despite having detected a face in the image (i.e., “classification failed”) (Noldus, 2021). Instances of “detection failed” occur when the face moves outside the visible area or in cases of very extreme (self-)occlusions (e.g., hands fully covering the face or the eyes) (Noldus, personal communication, March 16, 2023). To reduce the likelihood of “detection failed”, the face localization algorithm was set to search for a face size ranging from very small (.01 fraction of the image) to very large (1.0 the image fraction) (Noldus, 2021). “Classification failed” is output whenever the certainty of the deep neural network model fit falls below the minimum model certainty threshold – here, the default threshold of .50 on a scale between 0 (low model fit certainty) to 1 (high model fit certainty) (Noldus, 2021). Instances of “classification failed” occur in frames with 1) poor image quality, 2) extreme head poses (exceeding ± 30º angle in roll, yaw, pitch), 3) significant occlusions of the face (exceeding 25% of the face) or key facial features (e.g., the eyes, most of the mouth) (Noldus, personal communication, March 16, 2023). Figure 3 presents frames with partial facial occlusions for which the automated action unit detection failed. Time samples in which Baby FaceReader 9 modeled the facial expression successfully but were classified as “not visible” by the manual coder were rare (< 0.01% of the total manually coded data; Table 3) and were removed from further analyses.

**Table 3:** Automatically analyzed and missing data (classification failed) per manually coded facial expression category

Manual code	Automated analysis	Overall	4-month wave	8-month wave
Total 508,950 (100%)	Automatically analyzed	431,036 (85%)	253,018 (94%)	178,018 (74%)
	Classification failed	77,914 (15%)	16,186 (6%)	61,728 (26%)
	Total	508,950 (100%)	269,204 (100%)	239,746 (100%)
Positive 145,153 (29%)	Automatically analyzed	133,207 (92%)	68,585 (97%)	64,622 (87%)
	Classification failed	11,946 (8%)	1989 (3%)	9957 (13%)
	Total	145,153 (100%)	70,574 (100%)	74,579 (100%)
Neutral 337,119 (66%)	Automatically analyzed	275,449 (72%)	172,409 (93%)	103,040 (68%)
	Classification failed	61,670 (18%)	12,999 (7%)	48,671 (32%)
	Total	337,119 (100%)	185,408 (100%)	151,711 (100%)
Negative 24,662 (< 5%)	Automatically analyzed	21,676 (88%)	11,471 (94%)	10,205 (72%)
	Classification failed	2986 (12%)	709 (6%)	2,277 (18%)
	Total	24,662 (100%)	12,180 (100%)	12,482 (100%)
Not visible 2,016 (< 1%)	Automatically analyzed	704 (35%)	553 (53%)	151 (16%)
	Classification failed	1312 (65%)	489 (47%)	823 (84%)
	Total	2016 (100%)	1042 (100%)	974 (100%)
Total excluding not visible 506,934 (> 99%)	Automatically analyzed	430,332 (85%)	252,465 (94%)	177,867 (74%)
	Classification failed	76,602 (15%)	15,697 (6%)	60,905 (26%)
	Total	506,934 (100%)	268,162 (100%)	238,772 (100%)

*Note.* Counts refer to time samples (15 Hz, i.e., 0.067 s). The Manual Code column lists the total number of time samples for each manual code and as a percentage from the total available data. A total of 430,332 (85%) automatically analyzed time samples after excluding “not visible” were used for the main analysis. For each Manual Code category, percentages refer to the available and missing data from the category total

### Statistical analyses

To match the output rate of Baby FaceReader 9, the manually coded datastream was downsampled from 30 to 15 Hz (0.067 s). Unless otherwise specified, a 0.067-s epoch is the unit of analysis throughout the manuscript. Whereas higher temporal resolutions are necessary for applications involving real-time measurement or discrimination of muscle motion phases (Mavadati et al., 2013; Polikovsky et al., 2013), a 15-Hz measurement is sufficient for reliable offline detection of affective facial expressions. Further details regarding data synchronization can be found in Supplementary Materials A (https://osf.io/5zp2g).

All statistical analyses were performed in RStudio (v2022.07.1, R Core Team, 2022). The study hypotheses and analysis plan were pre-registered at the Open Science Framework (OSF) platform: https://osf.io/hrw8k/?view_only=111816181ea5488bb373e8b6f5f3ab38. The analysis plan includes the anonymized data and the data pre-processing analysis scripts, which are made available on the project’s GitHub repository: https://github.com/MZaharieva/Baby_FaceReader9_Validation.

#### Automated-manual vs. manual-manual classification accuracy

To quantify the accuracy with which Baby FaceReader 9 classifies manually coded facial expressions, we used the receiver operating characteristic (ROC) curve analysis implemented in the R-package “pROC” (v. 1.18.0; Robin et al., 2011). A high-accuracy measurement system maximizes the rate of correct classifications (i.e., the true-positive and true-negative rate) while minimizing incorrect classifications (i.e., the false-positive and false-negative rate). We treated the manual coding as the ground truth relative to which we estimated the probability of the automated system correctly predicting the presence and absence of a given affective facial expression. We reported several agreement metrics quantifying the trade-off between correct and incorrect classifications for discriminating between each manually coded facial expression pair (Girard et al. (2015): Area Under the ROC curve (AUC), positive agreement (PA, which is equivalent to F1 for binary classification problems), and negative agreement (NA). AUC is a single parameter summarizing the degree of discriminability between any two facial expression categories across all possible combinations of sensitivity and specificity (Clarke & Gilks, 2010). AUC is a threshold-independent metric of classification accuracy that is robust to imbalanced data (Jeni et al., 2013). PA quantifies automated-manual agreement for correct classifications (i.e., between-system agreement on the presence of a given ground-truth identified affective facial expression) by weighing the true positive rate against the misclassification rate: $\frac{2\times tp}{2\times tp+fp+fn}$ (Altman, 1990). NA – the complement of PA, quantifies automated-manual agreement for correct rejections (i.e., between-system agreement on the absence of a given ground-truth identified affective facial expression) by weighing the true negative rate against the misclassification rate: $\frac{2\times tn}{2\times tn+fp+fn}$ (American Psychiatric Association, 1994). PA and NA are sensitive to imbalanced data – with PA consistently underestimating correct classification rate (Jeni et al., 2013) – which is relevant for evaluating the performance of Baby FaceReader 9 for predicting the presence of affective facial expressions that are less frequently encountered in the current dataset.

We performed a multi-class ROC analysis, in which we quantified the degree of discriminability for each manually coded facial expression pair. This allowed us to identify specific pairs of manually coded facial expressions for which the distributions of automatically detected valence were separable even when not all three facial expression categories were separable. To assess whether the concordance among the manual and automated systems was comparable to that observed among two manual coders, we ran the same multi-class ROC analysis on a subset of the data with another independent manual coder as the predictor of manually coded facial expression category.

The effects of infant- and video-specific characteristics – infant age, out-of-plane head rotations, interaction partner, and face model fit certainty – on classification accuracy were explored in a series of multilevel regression models, in which the variability of the video-level AUC summary statistic nested within infants was treated as the outcome. Video-level AUC scores were derived by fitting two binary ROC curves for each video, quantifying the classification accuracy at which the automatically detected valence distinguished: 1) positive from negative and neutral manually coded facial expressions, and 2) negative from neutral manually coded facial expressions. Video-level AUC scores were further used to assess classification performance of Baby FaceReader 9’s global valence formula and AU12 at the video-level.

As robustness checks, we repeated the multi-ROC analysis using 1) the raw action unit output, replicating the results reported using the action unit output derived with temporal smoothing (available on GitHub), 2) a split dataset comparing classification accuracy for head rotation angles within ±20º versus head rotation angles of ±20º-30º, largely replicating the results reported (Table S11 [https://osf.io/43zqv]; Supplementary Materials C [https://osf.io/7wv3p]).

### Action unit activation intensity for positive versus negative facial expressions

We explored the relations between manually coded facial expression categories and the activation intensities of automatically detected action unit configurations that have been reported to be associated with positive and negative facial expressions (Table 1). In a Bayesian framework, we fit a multilevel multinomial logistic regression with a participant-level random intercept in the R-package "brms" (v.2.16.1; Bürkner, 2017, 2018) at the 15-Hz time sample level using a priori action unit configurations to predict the probability of a manually coded facial expression being either positive, negative, or neutral as the reference category. Whenever convergence issues arose, the fixed effects were evaluated in a model without a random intercept. The probability of the intensity of the action unit configurations hypothesized to indicate positive versus negative facial expressions (Table 1) being assigned to either manually coded category was formulated as follows:

$$logit\left(Manually\; Coded\; Facial\; Expression\; Category\right)={\beta }_{0}+{\beta }_{AU12}+{\beta }_{AU6}+{\beta }_{AU(25+26+27)}+{\beta }_{AU17}+{\beta }_{AU20}+{\beta }_{AU\left(3+4\right)}{+ \beta }_{AU\left(6+7\right)}+{\beta }_{AU12}\times {\beta }_{AU6}+{\beta }_{AU12}\times {\beta }_{AU(25+26+27)}+ {\beta }_{AU17}\times {\beta }_{AU\left(3+4\right)}+{\beta }_{AU20}\times {\beta }_{AU\left(3+4\right)}+{\beta }_{AU20}\times {\beta }_{AU\left(6+7\right)}+{ \beta }_{AU20}\times {\beta }_{AU(25+26+27)}$$

To evaluate the degree of uncertainty in the estimated parameter values in the hypothesized statistical model (Claeskens & Hjort, 2008), we performed parameter selection using Bayesian model averaging in the R-package “BAS” (v.1.6.0, Clyde & Clyde, 2015; for an overview, see van den Bergh et al., 2021, and Hinne et al., 2020).

In a post hoc analysis, we explored two parsimonious models using independent action units to predict positive and negative manually coded facial expressions. The intensity of smiling (AU12) alone was used to discriminate positive from negative or neutral manually coded facial expressions, whereas the intensities of lip stretching (AU20), and brow-lowering (AU3+AU4) were used to independently discriminate negative from neutral manually coded facial expressions.

## Results

### Face model fit certainty

The overall face model fit certainty of the automated measurement was acceptable (M = .62, SD = .05). Lower mean model fit certainty was observed for the manually coded "not visible" category than for other manual coded facial expression categories (M = .56, SD = .05) (Table S1 [https://osf.io/s6e5a] and Fig. S2 [https://osf.io/ea6bt]), and to a lesser extent – for infants at 4 compared to 8 months of age (Table S1 [https://osf.io/s6e5a] and Table S2 [https://osf.io/h85kb]). No substantial differences were observed across interaction partners (Table S1 [https://osf.io/s6e5a] and Table S2 [https://osf.io/h85kb]).

### Missing data comparison

Baby FaceReader 9 returned no instances of “detection failed”. Baby FaceReader 9 returned “classification failed” for 15% of the time samples (Table 3). A substantially larger percentage of “classification failed” was encountered in the automated measurement of the 8-month wave (26%) than the 4-month wave (6%), which was consistent across manually coded facial expression categories. Manual coders coded the face as “not visible” in fewer than 1% of samples (Table S4 [https://osf.io/wvyf9]). Baby FaceReader 9 failed to estimate action unit intensities (“classification failed”) for 65% of the time samples that were manually coded as "not visible". Time samples for which the automated face “classification failed” or were marked as “not visible” by the manual coders were removed from further analyses. The final dataset contained 430,332 time samples (0.067-s epochs) from 289 videos of 51 infants at 4 months and 53 infants at 8 months.

### Manual-manual classification accuracy

To establish the maximum classification accuracy that might be theoretically expected for the automated system, a multi-ROC model was fitted to the 15% of the videos that had been randomly selected to be coded by two independent manual raters. The results showed excellent mean AUC and PA scores for discriminating between all three facial expression categories: 1) AUC = .99 and PA = .99 for positive from negative, 2) AUC = .94 and PA = .92 for positive from neutral, and 3) AUC = .97 and PA = .99 for negative from neutral between the manual coders.

### Predicting manually coded facial expressions from automatically detected global emotional valence

#### Automatically detected valence distributions

Automatically detected valence values were more positive for facial expressions that were manually coded as positive than those coded as neutral or negative (Fig. S4 [https://osf.io/nj8qb.]). The automatically detected valence distributions of the facial expressions that were manually coded as negative and neutral were centered around zero and largely overlapped. This pattern was consistent across measurement waves (Fig. S4 [https://osf.io/nj8qb]) and interaction partners (Fig. S5 [https://osf.io/s7arp] and Table S5 [https://osf.io/qegdv]).

### Automated-manual classification accuracy using Baby FaceReader 9’s global emotional valence formula

A multi-class ROC analysis was performed on the 430,332 time samples using the automatically detected global emotional valence formula to discriminate between the three ordered manually coded facial expression categories: negative, neutral, and positive. Table 4 summarizes the PA and NA scores observed at the automatically detected valence value that yielded the greatest discriminability between positive, neutral, and negative manually coded facial expressions in the current dataset.

**Table 4:** Classification performance metrics of BabyFace Reader 9’s global emotional valence formula to discriminate between manually coded affective facial expressions

	Threshold	Sensitivity *i*, Specificity *j*	AUC	*PA (*F1)	NA
*Overall*					
Positive vs. Negative	.03	.74, .71	.79	.83	.43
Positive vs. Neutral	.07	.68, .84	.82	.68	.84
Negative vs. Neutral	– .04	.54, .52	.49	.69	.14
Positive vs. Negative/Neutral*	.07	.83, .68	.81	.84	.67
Negative vs. Neutral*	– .04	.54, .52	.58	.69	.14
*4 Months*					
Positive vs. Negative	.01	.75, .71	.80	.83	.45
Positive vs. Neutral	.07	.65, .85	.81	.64	.85
Negative vs. Neutral	– .02	.45, .64	.50	.61	.13
*8 Months*					
Positive vs. Negative	.03	.78, .66	.78	.85	.43
Positive vs. Neutral	.07	.71, 83	.82	.72	.83
Negative vs. Neutral	-.05	.65, .41	.49	.76	.17

*Note.* Threshold values are computed using Youden’s J statistic (Youden, 1950), which uses the distance to the ROC identity (diagonal) line to select a cut-off value that maximizes the specificity and sensitivity of automatically detected valence to discriminate each pair of manually coded facial expressions. Statistics are reported at the time-sample (15 Hz) level with the exception of contrasts marked with an asterisk *, which were computed at the video level

**Fig. 4:**
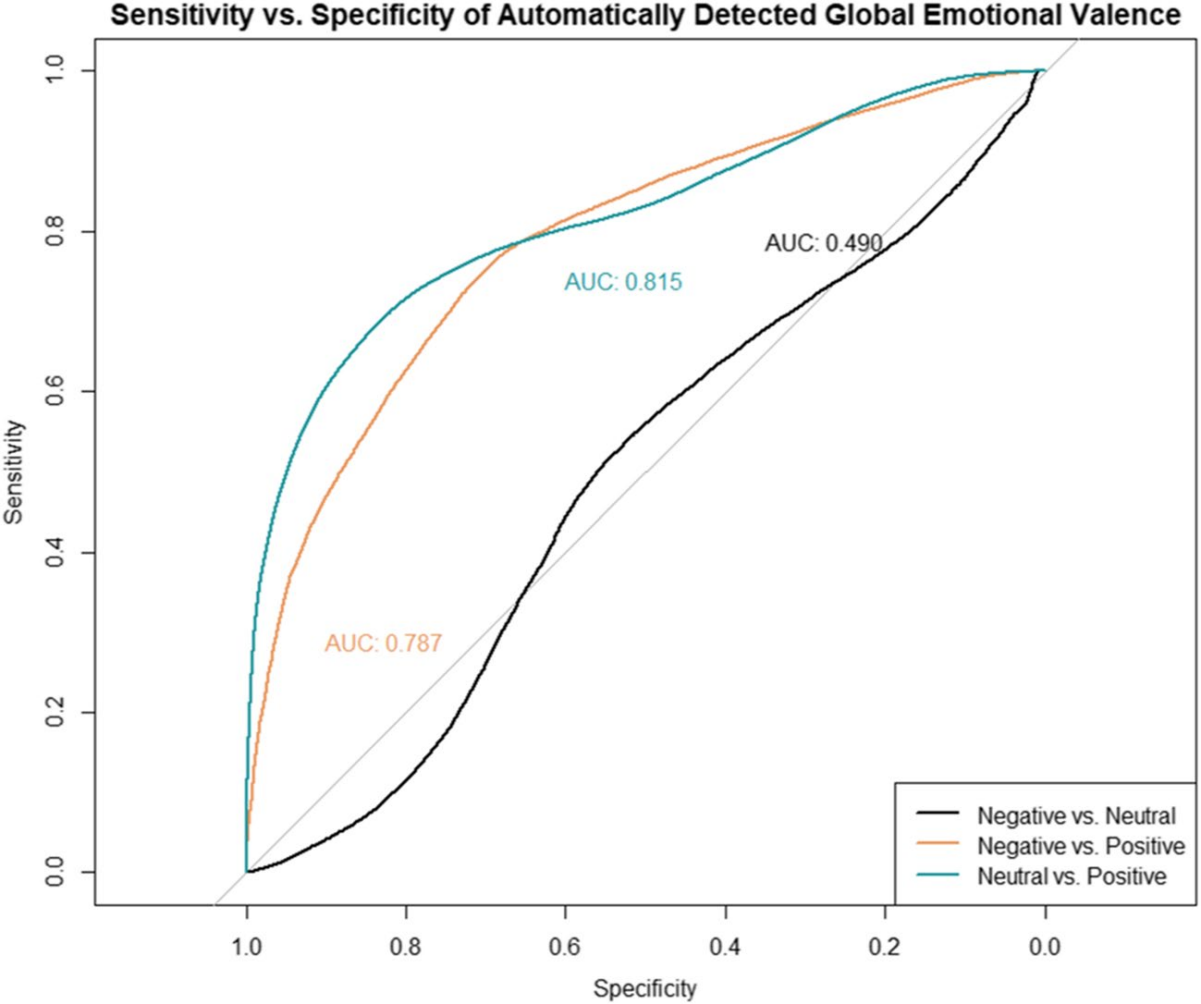
ROC functions describing the sensitivity and specificity at which Baby FaceReader 9’s global emotional valence formula discriminates between manually coded facial expressions. *Note.* The ROC functions describing the trade-off between sensitivity and specificity per manually coded facial expression category is plotted against chance-level classifier accuracy (gray line). The area under the curve (AUC) coefficient is displayed next to each ROC function

AUCs for the automatically detected valence indicated 79% chance of correctly distinguishing positive from negative manually coded facial expressions, and 82% chance of distinguishing between positive from neutral manually coded facial expressions (Fig. 4). Correct classifications (PA) of positive from negative manually coded facial expressions were consistently above .80, whereas correct rejections (NA) were below chance level (Table 4). Correct classifications of positive from neutral manually coded facial expressions were moderate (PA = .68), whereas correct rejections were low (NA = .84). Importantly, the discrimination of negative from neutral manually coded facial expressions was at chance level (AUC = .49; PA = .69; NA = .14). Similar results were obtained when individual ROC curves were fit to at the video level, thereby accounting for individual differences between infants and infant ages (Table 4; Supplementary Materials C [https://osf.io/7wv3p]).

### Effects of infant-specific and video-specific characteristics on classification accuracy

Infant age, interaction partner, horizontal and vertical out-of-plane head rotations, face model fit certainty, and the interactions among them were used to predict video-level classification accuracy (AUC scores); the detailed results are reported in Supplementary Materials A (https://osf.io/5zp2g). Higher face model fit certainty predicted higher classification accuracy in the model comparing positive versus other facial expressions (Table S7.A [https://osf.io/d52kb]). Horizontal (yaw) and vertical (pitch) head rotation angles were consistently higher for infants at 8 compared to 4 months of age (Table S6 [https://osf.io/vwr9d]; Fig. S6 [https://osf.io/9s5yg]; Supplementary Materials C [https://osf.io/7wv3p]). Consistent with previous automated action unit detection work in adults (Girard et al., 2015; Valstar et al., 2017), after accounting for infant age and face model certainty, the eccentricity of yaw and pitch head rotations within ± 30º showed small, negative correlations with mean video-level AUC scores (Table S9.A [https://osf.io/nc4ep] and Table S10.A [https://osf.io/cqfht]). The AUC scores for Baby FaceReader 9’s global emotional valence formula, smiling (AU12), brow lowering (AU3+AU4), and lip stretching (AU20) obtained for head rotations between ± 20 and 30º remained within 5% deviation of the AUCs obtained for head rotations within ± 20º (Table S11 [https://osf.io/43zqv]). Detailed results are reported in Supplementary Materials C [https://osf.io/7wv3p].

Taken together, Baby FaceReader 9’s global emotional valence formula showed moderate to high classification accuracy and misclassification rate when distinguishing positive from the combined set of negative or neutral manually coded facial expressions. However, the discrimination of negative from neutral facial expressions was at chance-level.

## Predicting manually coded facial expressions from a priori automatically detected action unit configurations

### Automatically detected action units distributions

**Fig. 5:**
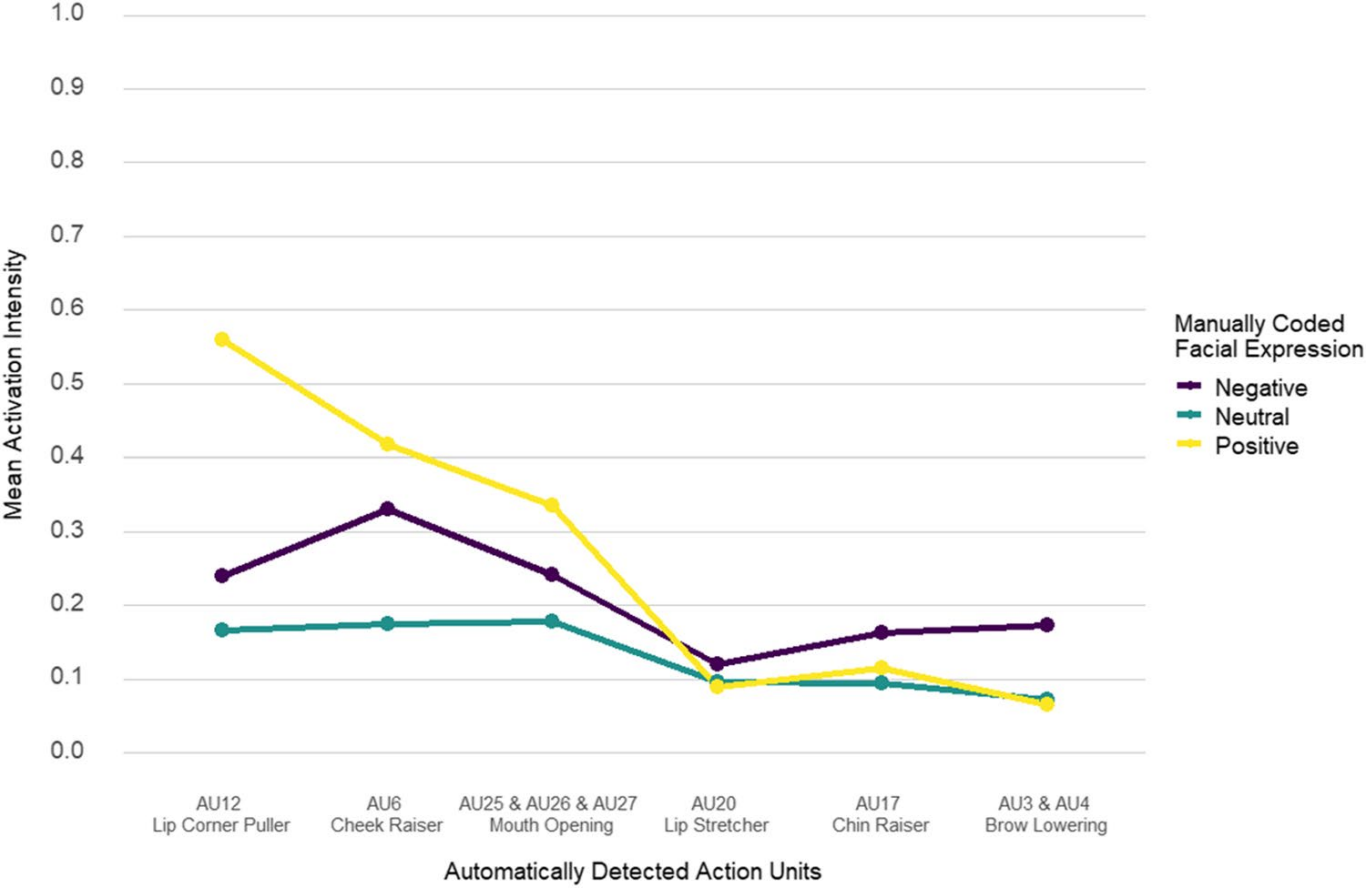
Mean action unit activation intensity per facial expression category**.** *Note.* Action unit intensity ranges between 0 (low) to 1 (high) corresponding to the intensity categories described in Baby FACS (Oster, 2006): “inactive” [.00 – .10], A “trace” [.10 – .22]; B “slight ” [.22 – .33]; C “pronounced” [.33 – .62]; D “severe” [.62 – .91]; E “max” [.91 – 1.00] (Noldus, 2021). Positive manual codes were characterized by higher mean intensity levels of lip corner raiser (AU12), cheek raiser (AU6), and lips parting (AU25) as opposed to negative or neutral manual codes. Brow lowering (AU3+AU4), lip stretching (AU20), cheek raiser (AU6), and lips parting (AU25) showed higher mean intensity levels for negative as opposed to neutral manual codes

The occurrence and intensity base rates of the automatically detected action units hypothesized to index positive and negative facial expressions are summarized in Table S12.A [https://osf.io/bwxzt] and Table S12.B [https://osf.io/by8uh], respectively. Automatically detected lip corner raiser (AU12), cheek raiser (AU6), and lips parting (AU25) was detected in over half of the video samples and showed “pronounced”-level mean activation intensity during positive manually coded facial expressions. Automatically detected brow lowering (AU3+AU4), lip stretching (AU20), and pouting (AU17) was detected in one-third of the video samples (or less) and showed “trace”-level mean activation intensity for negative manually coded facial expressions. As a consequence, assessing the classification performance of the a priori action unit model for distinguishing between negative and positive manually coded facial expressions was less reliable. Positive manual codes were characterized by higher mean intensity levels in the lip corner raiser (AU12), cheek raiser (AU6), and lips parting (AU25) than negative or neutral manual codes (Fig. 5). Brow lowering (AU3+AU4), lip stretching (AU20), cheek raiser (AU6), and lips parting (AU25) showed higher mean intensity levels for negative as opposed to neutral manual codes (Fig. 5). Lid tightener (AU7) showed low activation intensity across manually coded facial expression categories (Table S12.B [https://osf.io/by8uh]) and a relatively low correlation with cheek raiser (AU6) intensity (*r* = .23, *p* < .001). Hence, only the main effect of the cheek raiser (AU6) was used to indicate eye constriction in the subsequent logistic regression analysis.

### Discriminating positive, negative, and neutral facial expressions from a priori action unit configurations

Next, we assessed whether manually coded facial expressions could be predicted from automatically detected action unit combinations hypothesized to index the positive and negative facial expression configurations reported in Table 1. Because manually coded negative facial expressions were sparse in our data (< 5% of the manually coded data; Table S4 [https://osf.io/wvyf9.]), we fit a series of binary logistic regression models in a Bayesian framework using the a priori automatically detected action unit combinations to discriminate: 1) manually coded positive from negative and neutral facial expressions pooled into a single category (430,332 time samples from 289 videos), 2) manually coded neutral from negative facial expressions (297,125 time samples from 289 videos). To dampen the sample imbalance, the inverse label distributions of the outcome were assigned as weights in both regression models. We used Bayesian model averaging to re-estimate the logistic regression models as a robustness check and selected those parameters that systematically explained variance across the full model space (Hinne et al., 2020). The sum activation of lips parting (AU25), jaw dropping (AU26), and mouth stretching (AU27) was used as a coarse measure of mouth opening.

Detailed results are presented in Supplementary Materials D (https://osf.io/rgbqd.). The a priori action unit model for discriminating positive from negative facial expressions achieved high correct rejection rate (*NA* = .88), but low correct classification rate (*PA =* .66). Nevertheless, the hypothesized automatically detected action units were individually predictive of positive manually coded facial expressions rather than negative and neutral combined. Automatically detected smiles (AU12) were strongly associated with manually coded positive as opposed to negative or neutral facial expressions, as were, to a lesser extent, eye constriction (AU6) and mouth opening (AU25+AU26+AU27). The co-occurrence of smiling (AU12) with eye constriction (Duchenne smiling; AU12+AU6) or mouth opening (AU25+AU26+AU27) did not contribute strongly to the identification of positive manually coded facial expressions. The a priori action unit model for discriminating negative from neutral facial expressions achieved high correct rejection rate (*NA* = .92), but an even lower correct classification rate (*PA =* .38). Automatically detected lip stretching (AU20), brow lowering (AU3+AU4), eye constriction (AU6), mouth opening (AU25+AU26+AU27), and to a lesser extent – cry faces with eye constriction (AU20+AU6) were associated with negative rather than neutral manually coded expressions. The action unit configurations involving automatically detected pouting (AU17) and lip-stretching (AU20) with brow lowering (AU3+AU4) were indicative of neutral rather than negative manually coded facial expressions.

### Discriminating positive, negative, and neutral manually coded facial expressions using automated measurement of the intensity of individual action units

Next, we adopted a more parsimonious approach using individual automatically detected action units rather than action unit configurations to detect manually coded facial expressions. We used smiling (AU12) to detect positive facial expressions (Ekman et al., 2002; Messinger et al., 2001), and lip stretching (AU20) and brow lowering (AU3+AU4) to detect negative facial expressions (Matias & Cohn, 1993; Messinger et al., 2012; Oster, 2006; Oster & Rosenstein, 1996). Multi-class ROC analyses were performed on the 430,332 time samples using the individual activation intensities of AU12, AU3+AU4, and AU20 to discriminate between the three manually coded facial expression categories – positive, neutral, and negative.

**Fig. 6:**
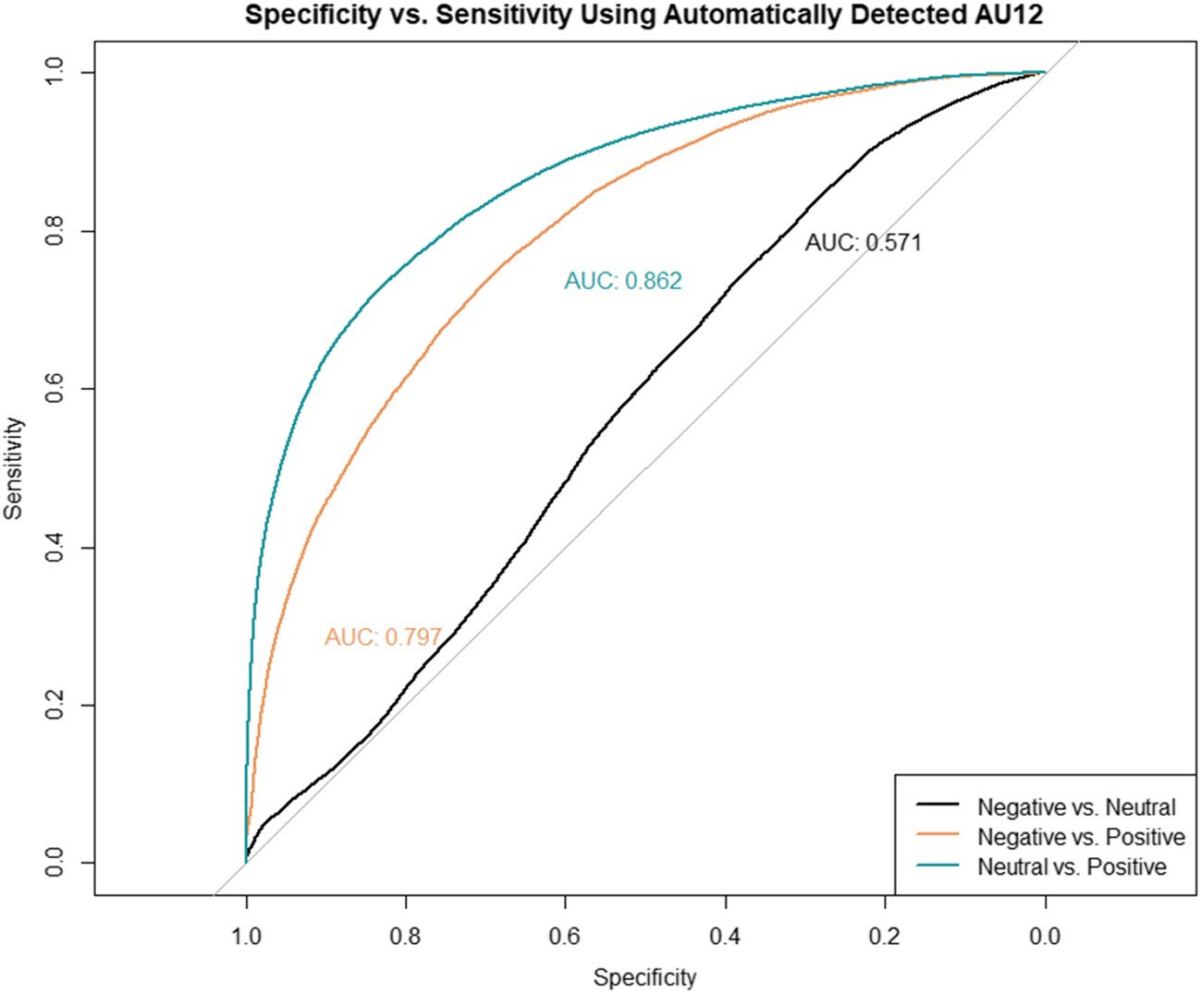
ROC functions describing the sensitivity and specificity at which the automatically detected smiling (AU12) discriminates between manually coded facial expressions *Note.* The ROC functions describing the trade-off between sensitivity and specificity per manually coded facial expression category is plotted against chance-level classifier accuracy (gray line). The area under the curve (AUC) coefficient is displayed next to each ROC function

The PA and NA scores observed at the automatically detected smile (AU12) intensity that yielded the greatest discriminability of positive from negative or neutral manually coded facial expressions are reported in Table 5; the ROC functions are plotted in Fig. 6. We observed 86% chance of correctly discriminating positive from negative/neutral facial expressions based on the AUC score (95% CI[.85 – .86]), and chance-level discrimination for negative from neutral (95% CI[.56 – .57]). The classification accuracy and misclassifications for discriminating positive from negative or neutral facial expressions based on AU12 alone was comparable to that using the global valence formula or the a priori smile configurations.

**Table 5:** Classification performance metrics of automatically detected smiling (AU12) to discriminate between manually coded facial expressions

	Threshold	Sensitivity *i*, Specificity *j*	AUC	*PA (*F1)	NA
*Overall*					
Positive vs. Negative	.23	.75, .69	.86	.83	.43
Positive vs. Neutral	.25	.73, .83	.80	.70	.85
Negative vs. Neutral	.26	.84, .29	.57	.89	.17
Positive vs. Negative/Neutral*	.25	.81, .73	.86	.84	.69
*4 Months*					
Positive vs. Negative	.21	.72, .75	.82	.82	.44
Positive vs. Neutral	.23	.70, .83	.85	.66	.85
Negative vs. Neutral	.18	.77, .30	.48	.85	.12
*8 Months*					
Positive vs. Negative	.24	.79, .61	.77	.85	.41
Positive vs. Neutral	.28	.76, .82	.87	.74	.83
Negative vs. Neutral	.31	.84, .35	.39	.88	.23

*Note.* Threshold values are computed using Youden’s J statistic (Youden, 1950), which uses the distance to the ROC identity (diagonal) line to select the cut-off value that maximizes the specificity and sensitivity of automatically detected smiling (AU12) to discriminate each pair of manually coded facial expressions. Statistics are reported at the time-sample level (15 Hz) with the exception of *, which were computed at the video level

The PA and NA scores for discriminating positive, neutral, and negative manually coded facial expressions using automatically detected brow lowering (AU3+AU4) and lip stretching (AU20) are reported in Table 6 and Table S16 [https://osf.io/hz3dw], respectively. Moderate classification accuracy and correct classification rate were observed when discriminating negative from neutral facial expressions using automatically detected lip stretching (AU20) (AUC = .70; PA = .80); however, correct rejections were below chance level (Table S16 [https://osf.io/hz3dw] and Fig. S8 [https://osf.io/dgrbn]). The classification accuracy (AUC) and correct classifications (PA) for discriminating negative from either neutral or positive facial expressions using automatically detected brow lowering (AU3+AU4) were consistently above .80 (Table 6 and 7); however, correct rejections (NA) were below chance level.

**Table 6:** Classification performance metrics of automatically detected brow lowering (AU3+AU4) to discriminate between manually coded facial expressions

	Threshold	Sensitivity *i*, Specificity *j*	AUC	*PA (*F1)	NA
*Overall*					
Positive vs. Negative	.07	.84, .68	.83	.89	.51
Positive vs. Neutral	.07	.84, .22	.53	.49	.34
Negative vs. Neutral	.08	.80, .67	.81	.87	.32
Negative vs. Neutral*	.08	.80, .64	.79	.87	.40
*4 Months*					
Positive vs. Negative	.08	.89, .74	.87	.92	.61
Positive vs. Neutral	.06	.72, .41	.58	.45	.54
Negative vs. Neutral	.09	.87, .68	.83	.92	.36
*8 Months*					
Positive vs. Negative	.07	.78, .62	.78	.85	.42
Positive vs. Neutral	.06	.64, .43	.46	.50	.52
Negative vs. Neutral	.07	.71, .73	.80	.82	.31

*Note.* Threshold values are computed using Youden’s J statistic (Youden, 1950), which uses the distance to the ROC identity (diagonal) line to select the cut-off value that maximizes the specificity and sensitivity of automatically detected brow lowering (AU3+AU4) to discriminate each pair of manually coded facial expressions. Statistics are reported at the time-sample level (15 Hz) with the exception of *, which were computed at the video level

**Fig. 7:**
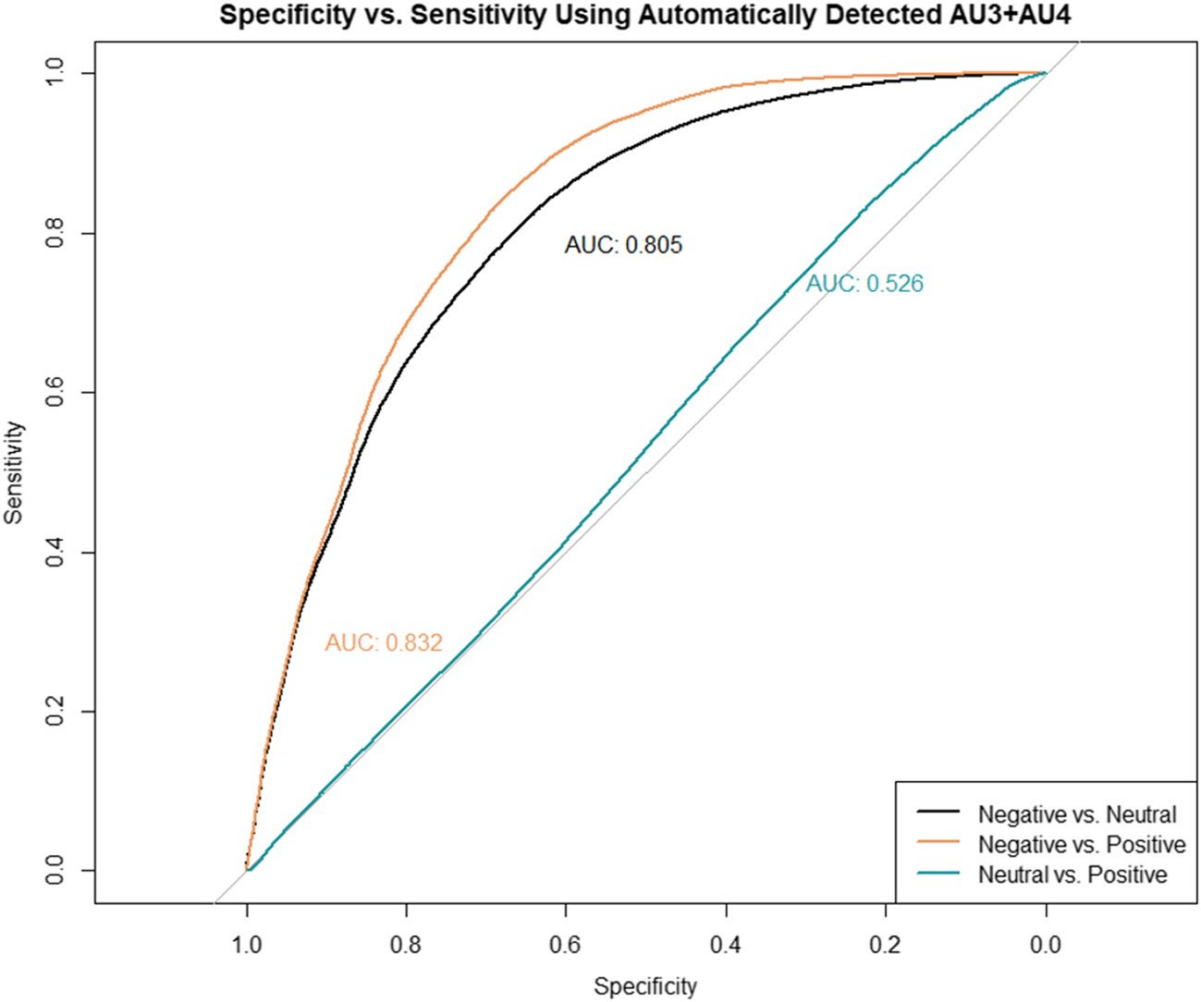
ROC functions describing the sensitivity and specificity at which the automatically detected brow lowering (AU3+AU4) discriminates between manually coded facial expressions. *Note.* The ROC functions describing the trade-off between sensitivity and specificity per manually coded facial expression category is plotted against chance-level classifier accuracy (gray line). The area under the curve (AUC) coefficient is displayed next to each ROC function

## Discussion

The time commitment involved in manual coding exerts a strong downward pressure on sample sizes, as well as the level of detail at which behaviors can be coded. To date, there have been only a handful of studies investigating infant affect using facial expressions in very large samples (e.g., Mitsven et al., 2022; Tronick et al., 2005). Over the past decade substantial progress has been made in developing automated techniques for measuring infant facial expressions (e.g., Hammal et al., 2017; Ertugrul et al., 2023; Messinger et al., 2012). Automated measurement via machine learning has the potential to be a time-efficient tool for classifying the global valence of infants’ facial expressions such as smiles and frowns. This could improve the replicability of infant studies by allowing researchers to apply objective measures to larger sample sizes than what is typically feasible with manual coding techniques. The current study assessed the validity and feasibility of a commercial system for automated facial expression measurement – Baby FaceReader 9 (Noldus, 2022), to discriminate between manually coded facial expressions in longitudinal data from infants at 4 and 8 months of age engaged in naturalistic face-to-face interactions with mother, father, and unfamiliar adult.

Though still well below the near-perfect agreement achieved between two manual coders on a small subset of the same data^1^, we found reasonable classification accuracy (AUC = .81) for distinguishing manual coding of positive from negative/neutral facial expressions at 4 and 8 months of age using the Baby FaceReader 9’s global emotional valence formula. However – in part due to the imbalanced samples – distinguishing manual coding of negative from neutral facial expressions was not reliable. Likewise, a set of pre-registered automatically detected action unit configurations that are central to the display of positive and negative affect (Ekman et al., 2002; Messinger et al., 2012; Oster et al., 1992, Oster, 2003, 2005a, b) was predictive of positive and negative manually coded facial expressions.

Whereas correct rejection rates were high, correct classification rates were only moderate for discriminating positive from negative/neutral manually coded facial expressions, and low for discriminating negative from neutral manually coded facial expressions. A parsimonious approach using only automatically detected smiling (AU12) reliably discriminated positive from negative or neutral facial expressions (AUC = .86). Importantly, automatically detected brow lowering (AU3+AU4) reliably distinguished negative from neutral facial expressions (AUC = .79). These results shed doubt on the implementation of complex a priori formulas in Baby FaceReader 9 (Noldus, 2022). However, results provide initial support for the automated detection of individual action units to recognize positive and negative facial expressions during naturalistic face-to-face interactions. Here we discuss the feasibility of using Baby FaceReader 9 to identify positive and negative facial expressions in young infants in terms of data availability, classification accuracy, and in comparison to alternative open-source tools, highlighting opportunities to improve automated system performance.

### Comparison of data availability

The application of fully automated measurement techniques to infant data is only feasible if it does not incur substantially greater data loss than that encountered with manual coding techniques. Overall, manual coding yielded fewer missing data (< 1% “not visible”) than the automated facial expression measurement via Baby FaceReader 9 (15%). Comparable or lower missing data rates have been reported for other automated action unit detection systems applied to face-to-face and still-face interactions in 4-month-olds infants (15% and 21%, respectively; Ahn et al., 2023), positive and negative interactions in 13-month-old infants (7% and 18%, respectively; Hammal et al., 2017), and for adult data with extreme head pose variations (0-33%; FERA 2017 Challenge; Valstar et al., 2017). Furthermore, Baby FaceReader 9 yielded a greater percentage of missing data for infants at 8 months (26%) than at 4 months (6%). Also in the successfully tracked data, consistently higher horizontal (yaw) and vertical (pitch) out-of-plane head rotation angles were observed for infants at 8 months compared to at 4 months. Given that manual coding yielded similar percentage missing data at both ages, the greater percentage missing data from the automated measurement in the older infants may reflect 8-month-olds’ increased mobility and tendency to make head-movements of greater eccentricity (Larson & Taulu, 2017). The automated measurement required a face angle within ± 30° pitch and yaw, whereas manual coders are able to capture changes in affective facial expressions in the presence of head movements as long as (parts of the) face are still visible.

A limitation of automated measurement using Baby FaceReader 9 is thus that it may yield more data loss than manual coding, particularly in data with extreme head pose variations. We recommend using a complementary approach in which data that cannot be coded automatically are coded manually, and, whenever appropriate – adopting data collection methods that minimize head movements. At the same time, the face resolution and, consequently – the face model fit certainty, were somewhat lower at 8 compared to 4 months. Whereas the current spatial resolution is likely sufficient for manual coding of discrete affective facial expressions across the whole face, obtaining video recordings at higher spatial resolutions may increase the chance of successful automated facial feature localization and tracking (i.e., of the facial landmarks specifying the location of relevant action units).

### Chance-level classification of sparse negative facial expressions at 4 and 8 months

Because of the positive nature of free-play interactions, negative facial expressions were sparse in our data, particularly at higher intensities (Mattson et al., 2013). Imbalanced samples bias statistical models in order to maximize classification performance for correctly predicting the most prevalent outcomes – neutral and positive manually coded facial expressions in the current dataset (Blagus & Lusa, 2010; Oommen et al., 2011). Perhaps as a result, the classification accuracy for discriminating negative from neutral facial expressions was hard to evaluate and at chance level. Fewer misclassifications can be expected in measurement contexts eliciting more frequent intense negative facial expressions. The assessment of classification accuracy thus requires further replication in a dataset where all three facial expression categories have more balanced distributions (Saito & Rehmsmeier, 2015). Suitable scenarios are for instance those invoking more frequent and intense negative affect displays such as Face-to-Face/Still-Face interactions (Tronick et al., 1978).

### Automated detection of positive facial expressions using Baby FaceReader 9’s global emotional valence formula

High classification accuracy (AUC), correct classifications (PA), and correct rejections (NA) are required for the classification performance of a predictor to be considered reliable. AUC quantifies classification accuracy over all potential trade-offs between sensitivity (correctly detecting the cases, e.g., positive) and specificity (correctly detecting the non-cases, e.g., neutral and negative). PA and NA complement each other to represent the trade-off between sensitivity (maximizing correct classifications and correct rejections) and precision (minimizing misclassifications, i.e., false positives and false negatives).

The video-level classification performance for discriminating manual codings of positive from negative/neutral facial expressions using Baby FaceReader 9’s global emotional valence formula was moderate to high (AUC = .81; PA = .84; NA = .67). These metrics are comparable to those reported for other automated facial expression analysis tools used to classify negative facial expressions in response to pain (for a review, Zamzmi et al., 2017) and looking behaviors (Chouinard et al., 2019; Hashemi et al., 2014). The moderate correct rejection rate may be problematic when applying an automated facial expression measurement system to unlabeled data from similar measurement contexts involving lower intensity facial expressions (e.g., face-to-face interactions). We recommend employing mixed approaches in which part of the data are both automatically and manually coded to establish inter-rater reliability.

### Predicting manually coded affective facial expressions from automatically detected action unit configurations

The intensity of both positive and negative facial expressions are characterized by several activation patterns involving functionally related facial muscles (Messinger et al., 2012; Oster et al., 1992; Oster, 2003, 2005a, b). Our results show that manual codings of positive and negative facial expressions were significantly predicted by a set of automatically detected action units that are central to the display of positive and negative affect (Ekman et al., 2002; Messinger et al., 2001). However, the logistic regressions that generated these predictions did not reliably classify the manually coded facial expressions.

Specifically, higher activation intensities of individual facial actions indexing smiling (AU12), eye constriction (AU6), and mouth opening (AU25+AU26+AU27) were associated with greater odds of manually coded positive rather than neutral or negative facial expressions. With respect to interaction effects, play smiling involving mouth opening (AU25+AU26+AU27) - but not Duchenne smiling involving eye constriction (AU6) - was weakly associated with greater odds of a manually coded positive expression. As less than 5% of the data were manually coded as negative, the action units thought to index negative affective expressions showed very low activation in our dataset and stable parameters could not be estimated for some effects. Automatically detected lip stretching (AU20), brow lowering (AU3+AU4), eye constriction (AU6), mouth opening (AU25+AU26+AU27), and to a lesser degree – Duchenne cry-faces involving lip stretching (AU20) with eye constriction (AU6), were predictive of manually coded negative rather than neutral facial expressions. Surprisingly, however, brow lowering (AU3+AU4) that occurred in combination with pouting (AU17) or lip stretching (AU20) was strongly predictive of neutral rather than negative manually coded facial expressions.

The individual effects of eye constriction (AU6) and mouth opening (AU25+AU26+AU27) in predicting positive and negative facial expressions are consistent with previous research demonstrating that these action units intensify both positive and negative affective displays (Mattson et al., 2013; Messinger et al., 2001, 2012; Ertugrul et al., 2023). However, the weak interaction effects of eye constriction (AU6) and mouth opening (AU25+AU26+AU27) in configuration with smiling (AU12) or lip stretching (AU20) suggest that the combination of these facial actions rarely exceeded their individual contributions. Taken together, these results suggest that complex a priori formulae involving multiple combinations of facial actions detected by Baby FaceReader 9 are not optimal tools for distinguishing manually coded positive and negative facial expressions.

High classification accuracy and correct classification rate (AUC = .86; PA = .84) but moderate correct rejection rate (NA = .69) was achieved using a parsimonious post-hoc approach based only on automatically detected smiling (AU12) to discriminate positive from negative/neutral facial expressions. The classification accuracy and correct classification rates for discriminating negative from neutral manually coded facial expressions based on automatically detected brow lowering (AU3+AU4) alone were also high (AUC = .79). The classification performance for discriminating negative from neutral manually coded facial expressions using automatically detected lip stretching (AU20) – a facial action muscle that is central to the infant cry-face (Messinger et al., 2012; Yale et al., 2003) – was moderate (AUC = .70). These results provide a promising case for the application of Baby FaceReader 9’s AU12 and AU3+AU4 (and perhaps AU20) detectors to discriminate positive from negative facial expressions during the face-to-face interactions of young infants.

Taken together, the classification of positive and negative manually coded expressions using Baby FaceReader 9 was superior based on the activation of individual action units rather than action unit configurations. To assist researchers interested in applying AU12 as an index of a social smile and AU3+AU4 and AU20 as an index of negative affect to their own data, we provide the automatically detected threshold values that yielded the best sensitivity and specificity levels at discriminating positive, neutral, and negative facial expressions in the current dataset (Tables 5 and 6, respectively). Further work is required to improve the mapping between negative affect and automatically detected action unit configurations, particularly for manifestations of pouting (AU17) and lip stretching (AU20) in combination with brow lowering (AU3+AU4).

### Previous work on automatic action unit detection

Previous work using open-source tools evaluated the concurrent validity of automatically detected action unit occurrences using manual Baby FACS-certified coding of action unit occurrence as ground truth (Ertugrul et al., 2023; Hammal et al., 2017). By contrast, we evaluated the construct validity of Baby FaceReader 9’s automatically detected action unit intensities using manual coding of global affective facial expression categories (positive, neutral, and negative) as ground truth. The differences in analytic approaches and ground truths preclude us from making a direct between-system comparison of Baby FaceReader 9 (Noldus, 2022), Infant AFAR (Ertugrul et al., 2023), and Hammal et al. (2017). For reference, we provide Table S17 (https://osf.io/pbj69) summarizing key performance metrics obtained in current and previous research on Baby FaceReader 9 (Noldus, 2022), Infant AFAR (Ertugrul et al., 2023), and Hammal et al. (2017). In future work, the continuous re-evaluation of concurrent and construct validity can be integrated into the lifecycle of automated action unit detection systems to allow for direct between-system performance comparisons, preferably using the same dataset(s) and analytic strategy across diverse measurement contexts (e.g., as done in the FERA 2017 challenge; Valstar et al., 2017).

### Cross-database generalizability

The deep convolutional neural network (CNN) model underlying Baby FaceReader 9 was trained for spontaneous action unit occurrence detection and intensity estimation on a compilation of real and augmented, predominantly frontal image data collected from infants between 6 and 24 months under relatively controlled, well-illuminated experimental conditions (Gudi et al., 2015; Noldus, 2021; Noldus, personal communication, March 8, 2023). The current study presents a case of cross-database model generalizability to an unseen dataset including wide-lens recordings of spontaneous facial expressions from younger infants (4 and 8 months), sparse negative affect displays, considerable head movement, and untrained recording conditions from home visits with variable illumination settings and recording angles. Importantly, we applied the model to a more general classification problem than the one it was originally trained for: predicting the semantic significance (e.g., positive, negative, neutral) of infant facial expressions. Cross-database model performance is expected to be poorer than model performance on unseen parts of the training dataset or datasets that more closely resemble the training dataset (Ertugrul et al., 2023). The robustness of automated action unit detection systems such as Baby FaceReader 9 could be improved if training datasets included greater variability in infant-specific characteristics (e.g., ages, ethnic backgrounds, facial anatomy), video-specific characteristics (e.g., lens angles, recording angles, recording sensors, resolution, illumination), and measurement contexts.

## Recommendations for future automated facial expression measurement applications in young infant samples

### Improving automated and manual facial expression measurement techniques

Facial muscle movement may be harder to detect (automatically) in younger infants who have higher levels of subcutaneous fat and less pronounced facial features (Oster, 2006). Baby FaceReader 9 was trained on static image data of infants between 6 and 24 months of age (Noldus, 2022), potentially making the model less readily generalizable to the appearance of action unit activation produced by younger infants. A straightforward solution for improving the classification accuracy is to train the Baby model of FaceReader 9 on a wider range of benchmark datasets from younger and more diverse infants and measurement contexts. Publicly available datasets are scarce (but cf. Messinger, 2014; Nanni et al., 2010; Webb et al., 2018) and it is important that the infant research community creates appropriate platforms for making such datasets publicly accessible (e.g., *Databrary*, Simon et al., 2015). Furthermore, extending Baby FaceReader with calibration features may offer more precision in automated action unit detection and tracking by accounting for individual differences in facial anatomy (but would involve collecting data during which the infant is not expressing any affect).

Here we considered manual coders, whose task was to categorize facial expressions as either positive, negative, or neutral – as the ground truth relative to which we assessed the automated system’s performance. However, it is not uncommon for infants in the first year of life to express components of positive as well as negative affect simultaneously (e.g., Adamson & Frick, 2003; Messinger et al., 1997; Weinberg & Tronick, 1996), and such expressions of mixed affect are of scientific interest. Thus, a more complete between-system performance comparison would be to build upon Baby FaceReader 9’s construct validity for classifying manually coded affective facial expressions (e.g., positive and negative) with manually coded Baby FACS data (Oster, 2006), in a similar fashion to that of Ertugrul et al. (2023), Maroulis et al. (2017), and Maroulis, 2018). The benefit of this approach is that the FACS coding system makes no reference to affective states (Cohn et al., 2007) such that concurrent validity can be estimated based on the between-system concordance of facial actions alone.

Although human observers – trained or untrained – are generally good at subjectively judging affective facial expressions in adults (Baker et al., 2010), infants do not necessarily express affective states in the same fashion as adults do (Camras et al., 2003; Kohut et al., 2012; Oster, 2006). Valid automated measurement of infant affective facial expressions, however, relies on understanding and formalizing the mapping between facial actions and their emotional significance. For instance, recognizing negative affect displays is particularly challenging because infants do not use a consistent set of action units when expressing lower intensity negative affect (Camras et al., 2003; Messinger et al., 2012). Systematic coding systems that explicitly operationalize possible variations in the morphology of affective facial behavior in early life – such as valid measurement of pouting mouth movements (AU17) involved in sad expressions – are thus essential for informing automated action unit detection algorithms and require continuous research.

### Collecting video data for automated facial expression analyses

The video data analyzed here were collected using a split-screen format with the intention that they be coded manually. This makes the current results broadly applicable to observational infant research using similar protocols. Nevertheless, improvements in classification accuracy and data availability of the automated measurement can be expected by taking additional steps to optimize video quality. Automated facial expression analyses can be sensitive to video image quality beyond spatial and temporal resolution (Beringer et al., 2019). Image brightness, sharpness, horizontal and vertical position of the light source with respect to the infant’s face, for example, have been previously related to the sensitivity of automated detection of gaze direction from image data (Chouinard et al., 2019). In the current dataset, Baby FaceReader 9’s face model fit certainty estimate – which reflects various video quality parameters – related to the classification accuracy. These results highlight the importance of assessing video recording conditions that may affect data availability and the accuracy of the automated measurement. We recommend that researchers pilot video recording devices and setups to increase automatic classification accuracy and reduce missing data.

## Conclusion

The study of real-time changes of infants’ affective facial expressions has been central to developmental science, enabling us to better understand a wide array of phenomena such as the development of emotion regulation (e.g., MacLean et al., 2014; Mangelsdorf et al., 1995) and preverbal communication (e.g., Beebe et al., 2016; Colonnesi et al., 2012; Hsu & Fogel, 2001; Yale et al., 2003). Moving toward automated behavioral measurement is important because it may permit detailed online and offline analysis of infant affect and communication with large sample sizes that are hard to obtain using standard manual coding techniques. The current study assessed the validity and feasibility of a turn-key instantiation for automated facial expression measurement - Baby FaceReader 9 (Noldus, 2022), to discriminate between manually coded facial expressions using longitudinal data from infants at 4 and 8 months of age engaged in naturalistic face-to-face interactions with mother, father, and unfamiliar adult. Our results shed doubt on complex a priori formulas, including Baby FaceReader 9’s global emotional valence formula, but provide initial support for the automated detection of individual action units to recognize positive and negative affect during naturalistic face-to-face interactions of infants as young as 4 months of age. Future work can profitably move toward improving automated measurement techniques to minimize data loss while identifying a priori action units and configurations that are central for the display of positive and negative affect.

## Data Availability

The study was pre-registered at the Open Science Framework platform (OSF): https://osf.io/hrw8k/?view_only=111816181ea5488bb373e8b6f5f3ab38. Fully anonymized data and documentation are available for public use and can be downloaded from GitHub: https://github.com/MZaharieva/Baby_FaceReader9_Validation or via the OSF repository. The raw video data used for the manual and automated facial expression analyses are stored in the data repository of University of Amsterdam and are not shared in open access format to protect participants’ confidentiality. For video data sharing requests, please contact Eliala A. Salvadori via eliala.salvadori@gmail.com.

## Footnotes

1 Manual-manual AUC, PA, and NA scores set the expected performance ceiling for the 1 current dataset.

## Code availability

All code for data cleaning and analyses associated with the current submission are provided as part of the GitHub and OSF repositories. Any updates will also be published on GitHub, and the final DOI will be cited in the manuscript.
